# Lot-to-lot immunogenicity consistency of the respiratory syncytial virus prefusion F protein vaccine in older adults

**DOI:** 10.1016/j.jvacx.2024.100494

**Published:** 2024-04-27

**Authors:** Murdo Ferguson, Alexander Murray, Lew Pliamm, Lars Rombo, Johan Sanmartin Berglund, Marie-Pierre David, Nathalie De Schrevel, Franck Maschino, Shady Kotb, Aurélie Olivier, Veronica Hulstrøm

**Affiliations:** a Colchester Research Group, 68 Robie, Truro, NS B2N 1L2, Canada; b PharmQuest, 806 Green Valley Rd Ste 305, Greensboro, NC 27408, United States; c Canadian Phase Onward Inc., Polyclinic Family and Specialty Medicine Facility, Polyclinic Family Health Group, 2 Champagne Dr, Toronto, ON M3J 0K2, Canada; d Clinical Research Centre Sörmland, Eskilstuna SE-631 88, Sweden; e Department of Medical Biochemistry and Microbiology, Zoonosis Science Center, Uppsala University, Uppsala SE-751 05, Sweden; f Department of Health, Blekinge Institute of Technology, Valhallavägen 1, Karlskrona SE-371 79, Sweden; g Department of Clinical Sciences, Lund University, BMC I12, Lund SE-221 84, Sweden; h GSK, Avenue Fleming 20, Wavre 1300, Belgium; i GSK, Rue de l'Institut 89, Rixensart 1330, Belgium

**Keywords:** Immunogenicity, Lot-to-lot consistency, Older adults, Prefusion F protein vaccine, Respiratory syncytial virus, Safety

## Abstract

**Background:**

Previous phase 3 studies showed that the AS01_E_-adjuvanted respiratory syncytial virus (RSV) prefusion F protein-based vaccine for older adults (RSVPreF3 OA) is well tolerated and efficacious in preventing RSV-associated lower respiratory tract disease in adults ≥ 60 years of age. This study evaluated lot-to-lot immunogenicity consistency, reactogenicity, and safety of three RSVPreF3 OA lots.

**Methods:**

This phase 3, multicenter, double-blind study randomized (1:1:1) participants ≥ 60 years of age to receive one of three RSVPreF3 OA lots. Serum RSVPreF3-binding immunoglobulin G (IgG) concentration was assessed at baseline and 30 days post-vaccination. Lot-to-lot consistency was demonstrated if the two-sided 95 % confidence intervals (CIs) of the RSVPreF3-binding IgG geometric mean concentration (GMC) ratios between each lot pair at 30 days post-vaccination were within 0.67 and 1.50. Solicited adverse events (AEs) within four days, unsolicited AEs within 30 days, and serious AEs (SAEs) and potential immune-mediated diseases within six months post-vaccination were recorded.

**Results:**

A total of 757 participants received RSVPreF3 OA, of whom 708 were included in the per-protocol set (234, 237, and 237 participants for each lot). Lot-to-lot consistency was demonstrated: GMC ratios were 1.06 (95 % CI: 0.94–1.21), 0.92 (0.81–1.04), and 0.87 (0.77–0.99) between the lot pairs (lot 1/2; 1/3; 2/3). For the three lots, the RSVPreF3-binding IgG concentration increased 11.84-, 11.29-, and 12.46-fold post-vaccination compared to baseline. The reporting rates of solicited and unsolicited AEs, SAEs, and potential immune-mediated diseases were balanced between lots. Twenty-one participants reported SAEs; one of these–a case of atrial fibrillation–was considered by the investigator as vaccine-related. SAEs with a fatal outcome were reported for four participants, none of which were considered by the investigator as vaccine-related.

**Conclusion:**

This study demonstrated lot-to-lot immunogenicity consistency of three RSVPreF3 OA vaccine lots and indicated that the vaccine had an acceptable safety profile.

ClinicalTrials.gov: NCT05059301.

## Introduction

Respiratory syncytial virus (RSV) is a contagious seasonal virus that causes respiratory tract infections in people of all ages. RSV infection usually causes a mild respiratory illness that resolves within 1–2 weeks [1], [2]. However, RSV can also cause more severe lower respiratory tract diseases (LRTDs), such as bronchitis, bronchiolitis, and pneumonia, that may require hospitalization. Although RSV is mostly recognized as a childhood illness [3], it also causes a significant disease burden in older adults [4], [5]. A recent meta-analysis estimated that 5.2 million cases of acute RSV infection occurred among adults ≥ 60 years of age in high-income countries in 2019, leading to 470,000 hospitalizations and 33,000 in-hospital deaths [5].

A progressive decline in immune function associated with aging, a phenomenon called immunosenescence, likely contributes to the increased susceptibility of older adults to RSV infection [6]. RSV can also cause more serious respiratory illnesses (including LRTDs) in older adults, especially in people with chronic medical conditions or those who are immunocompromised [4], [5]. RSV vaccination may therefore help reduce the RSV burden in this vulnerable population [7]. The AS01_E_-adjuvanted RSV prefusion F protein-based vaccine for older adults (RSVPreF3 OA, *Arexvy*, GSK) was first approved for protection against LRTD caused by RSV in adults ≥ 60 years of age in the United States (U.S.). To date, the vaccine is also approved in the European Union and other countries [8], [9], [10], [11], [12]. The vaccine contains a recombinant subunit prefusion RSV F glycoprotein antigen [13], combined with the liposome-based AS01_E_ adjuvant that improves the magnitude of the immune response [14], [15]. Results of an ongoing placebo-controlled phase 3 efficacy study demonstrated that RSVPreF3 OA was efficacious and well tolerated during two full RSV seasons in the northern hemisphere in adults ≥ 60 years of age, including people with underlying medical conditions [16], [17], [18]. In another ongoing phase 3 study, vaccination with RSVPreF3 OA was shown to induce a robust immune response, with humoral and cell-mediated responses persisting at least one year after a single dose [19].

For regulatory approval, manufacturing consistency must be demonstrated as per the World Health Organization requirements for vaccine development and pre-qualification [20], [21], [22]. In this phase 3 study, we assessed lot-to-lot consistency of three lots of RSVPreF3 OA in terms of immunogenicity and evaluated the reactogenicity and safety of one dose of each of these lots.

**Fig. 1:**
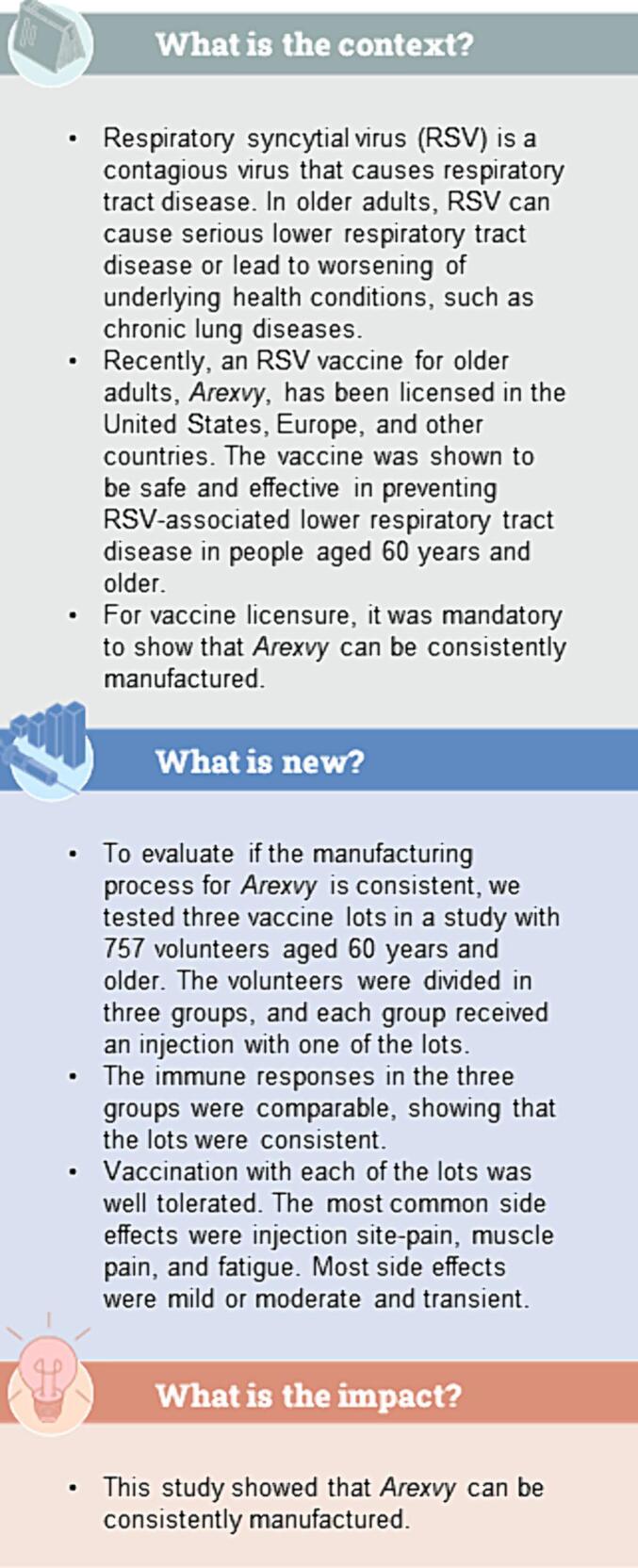
Plain language summary.

A plain language summary contextualizing the results of this study is presented in Fig. 1.

## Methods

### Study objectives

The primary objective of the study was to demonstrate lot-to-lot consistency in terms of immunogenicity of three lots of RSVPreF3 OA, as evaluated by RSVPreF3-binding immunoglobulin G (IgG) geometric mean concentration (GMC) ratios 30 days post-vaccination for each pair of the vaccine lots (i.e., lot 1/lot 2; lot 1/lot 3; lot 2/lot 3). Secondary objectives were to characterize the RSVPreF3-binding IgG responses of the three lots by means of GMCs at baseline and 30 days post-vaccination and mean geometric increases (MGIs), and to evaluate the vaccine’s reactogenicity and safety profile up to six months post-vaccination.

### Study design, participants, and conduct

This phase 3, randomized, double-blind study was conducted between 1 October 2021 and 30 June 2022 at 19 centers in three countries: seven in Canada, three in Sweden, and nine in the U.S. Participants were men and women aged ≥ 60 years at the time of study vaccination who, in the investigators’ opinion, could and would comply with protocol requirements, and who provided informed consent before any study-specific procedure. Individuals with chronic medical conditions, with/without specific treatment, were eligible if the investigator considered the participant’s condition to be medically stable. See Supplementary methods for the full list of inclusion and exclusion criteria.

The study was conducted according to the Declaration of Helsinki, the Council for International Organizations of Medical Sciences International Ethical guidelines, the International Council for Harmonization Good Clinical Practice guidelines, and applicable laws and regulations. The study documents were approved by national, regional, or study center independent ethics committees or institutional review boards. The protocol can be accessed at https://www.gsk-studyregister.com/en/trial-details/?id=217131. The study was registered at ClinicalTrials.gov (NCT05059301).

### Study intervention, randomization, and blinding

One 0.5 mL dose of RSVPreF3 OA consisted of 120 µg RSVPreF3 antigen and the liposome-based AS01_E_ adjuvant, comprising 25 µg of 3-*O*-desacyl-4′-monophosphoryl lipid A and 25 µg of *Quillaja saponaria* Molina, fraction 21. The vaccine was administered by intramuscular injection in the deltoid of the non-dominant arm.

The study participants were randomized (1:1:1) in three groups, to receive one of three lots of RSVPreF3 OA, each composed of unique combinations of antigen and adjuvant lots. More information on randomization is provided in the Supplementary methods.

The study was conducted in a double-blind manner until the time of the 30 days post-vaccination analysis timepoint of the final analysis of the primary objective) and in a single-blind manner thereafter, with study participants remaining blinded until study end (approximately six months post-vaccination).

### Immunogenicity evaluation

Blood samples were collected from participants at baseline (day 1, before vaccination) and 30 days post-vaccination (day 31). Serum RSVPreF3-binding IgG concentrations were assessed using an in-house enzyme-linked immunosorbent assay (ELISA) [15], [16], with a lower limit of quantification (LLOQ) of 25 ELISA units (EU)/mL and an upper limit of quantification (ULOQ) of 251,769 EU/mL.

### Reactogenicity and safety evaluation

Solicited administration-site adverse events (AEs) (pain, erythema, and swelling) and systemic AEs (fever, headache, myalgia, arthralgia, and fatigue) were recorded by the participants up to four days post-vaccination, and unsolicited AEs were recorded up to 30 days post-vaccination in paper diaries. Serious AEs (SAEs) and potential immune-mediated diseases (pIMDs) were recorded throughout the study, with a last safety contact approximately six months post-vaccination (study end).

### Statistical analyses

Statistical analyses were performed using SAS Life Science Analytics Framework. Primary and secondary immunogenicity objectives were analyzed on the per-protocol set (PPS) (all eligible participants who received the study intervention as per protocol, had immunogenicity results pre- and post-vaccination, complied with blood draw intervals, had no intercurrent conditions that could interfere with immunogenicity and did not receive prohibited concomitant medication/vaccination).

Approximately 750 participants were planned to be enrolled in the study (250 per vaccine lot group), to reach 225 participants per group in the PPS that would be evaluable for the primary objective (assuming a 10 % non-evaluable rate up to 30 days post-vaccination).

To assess lot-to-lot consistency, the two-sided 95 % confidence intervals (CIs) of the RSVPreF3-binding IgG GMC ratios at 30 days post-vaccination were calculated for each pair of RSVPreF3 OA vaccine lot groups (group 1/group 2, group 1/group 3, and group 2/group 3), using an analysis of covariance model on the log_10_-transformed concentrations that included the vaccine lot group and the age category (60–69 years, 70–79 years, or ≥ 80 years) as fixed effects, and the baseline concentration as covariate. As success criteria for equivalence of the three lots, the two-sided 95 % CIs of the group GMC ratios between each lot pair had to be within the pre-defined limits of 0.67 and 1.50.

RSVPreF3-binding IgG GMCs were calculated with 95 % CIs. The MGI, i.e., geometric mean of ratios of RSVPreF3-binding IgG concentrations 30 days post-vaccination over baseline, were calculated with 95 % CIs. Concentrations below the LLOQ were replaced by half the LLOQ, and concentrations above the ULOQ were replaced by the ULOQ. Missing data were not replaced. Demographic characteristics, safety, and reactogenicity were assessed on the exposed set (all participants who received the vaccine) and were summarized using descriptive statistics.

## Results

### Study population

**Fig. 2:**
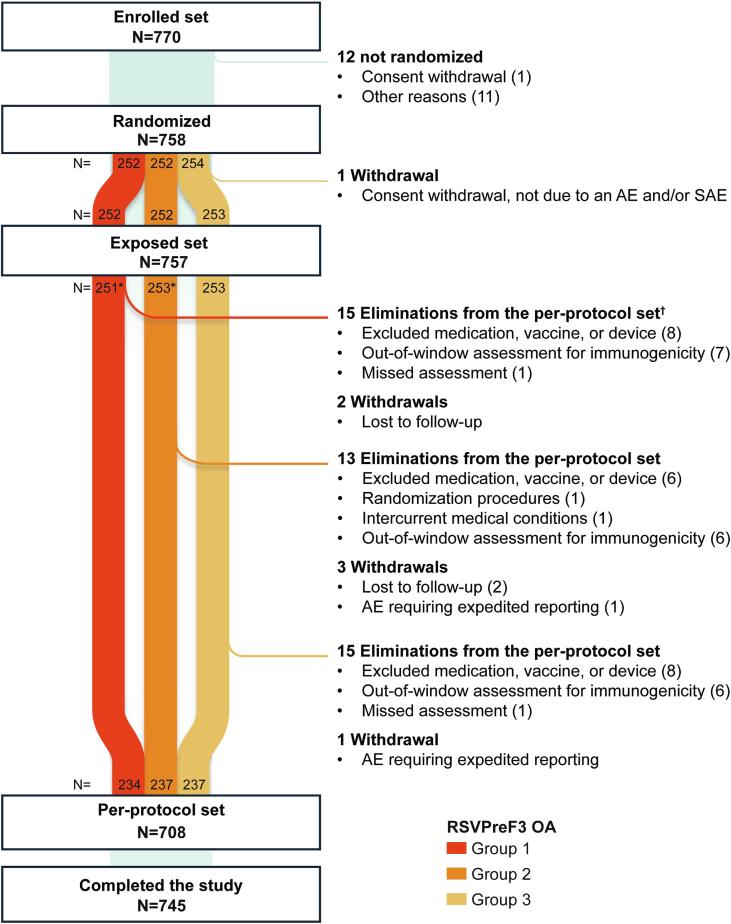
Flow of participants. N, number of participants; AE, adverse event; SAE, serious AE. *After randomization, one participant randomized into group 1 was incorrectly administered the vaccine lot of group 2, and therefore moved to group 2. †Participants could be eliminated from the per-protocol set for more than one reason. Note: Participants were considered to have completed the study if they returned or were available for the safety contact visit six months post-vaccination.

A total of 770 participants were enrolled, of whom 758 were randomized in the three vaccine lot groups and 757 received RSVPreF3 OA (251 in group 1, 253 in group 2, and 253 in group 3) (exposed set). Of these, 708 were included in the PPS (234 in group 1, 237 in group 2, and 237 in group 3). A total of 745 participants completed the study (six months post-vaccination safety contact) (Fig. 2).

Of the 757 participants in the exposed set, 49.0 % were female. Most participants were not Hispanic or Latino by ethnicity (96.6 %) and white by race (91.8 %). The mean age of the participants was 69.9 (±6.6) years. Overall, the demographic and baseline characteristics of the participants were comparable in the three vaccine lot groups (Table 1).

**Table 1:** Demographic characteristics of study participants at baseline, exposed set.

**Characteristic**	**Group 1** **N = 251**	**Group 2** **N = 253**	**Group 3** **N = 253**	**Overall** **N = 757**
Age				
Mean ± SD (years)	69.7 ± 6.6	70.1 ± 6.6	69.9 ± 6.6	69.9 ± 6.6

Age distribution, n (%)				
60–69 years	137 (54.6)	137 (54.2)	136 (53.8)	410 (54.2)
70–79 years	90 (35.9)	90 (35.6)	91 (36.0)	271 (35.8)
≥80 years	24 (9.6)	26 (10.3)	26 (10.3)	76 (10.0)
Female sex, n (%)	131 (52.2)	131 (51.8)	109 (43.1)	371 (49.0)
Not Hispanic or Latino ethnicity, n (%)	244 (97.2)	244 (96.4)	243 (96.0)	731 (96.6)

Race, n (%)				
Asian	6 (2.4)	10 (4.0)	10 (4.0)	26 (3.4)
Black or African American	6 (2.4)	3 (1.2)	4 (1.6)	13 (1.7)
Native Hawaiian/other Pacific Islander	0 (0.0)	1 (0.4)	0 (0.0)	1 (0.1)
White	231 (92.0)	231 (91.3)	233 (92.1)	695 (91.8)
Other	8 (3.2)	8 (3.2)	6 (2.4)	22 (2.9)

Country, n (%)				
Canada	105 (41.8)	105 (41.5)	109 (43.1)	319 (42.1)
Sweden	83 (33.1)	83 (32.8)	83 (32.8)	249 (32.9)
United States	63 (25.1)	65 (25.7)	61 (24.1)	189 (25.0)

N, number of participants in the exposed set; n/%, number/percentage of participants in a given category; SD, standard deviation.

### Immunogenicity

**Fig. 3:**
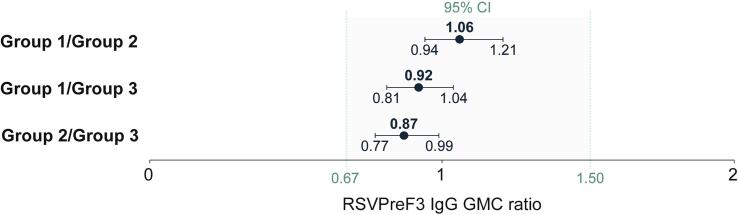
Lot-to-lot comparisons in terms of RSVPreF3-binding IgG GMC ratios between RSVPreF3 OA lot pairs at 30 days post-vaccination, per-protocol set. RSVPreF3, respiratory syncytial virus prefusion F protein; IgG, immunoglobulin G; GMC, geometric mean concentration; CI, confidence interval (depicted as error bars); EU, enzyme-linked immunosorbent assay unit. Note: The shaded area indicates the thresholds used to define lot-to-lot consistency (two-sided 95 % CIs of the group GMC ratios between each lot pair between 0.67 and 1.50 limits).

Thirty days post-vaccination, RSVPreF3-binding IgG GMC ratios between the RSVPreF3 OA lot pairs were 1.06 (95 % CI: 0.94–1.21) (group 1/group 2), 0.92 (0.81–1.04) (group 1/group 3), and 0.87 (0.77–0.99) (group 2/group 3), demonstrating lot-to-lot consistency, i.e., the two-sided 95 % CIs of the GMC ratios between each lot pair were between the pre-defined limits of 0.67 and 1.50 (Fig. 3).

At baseline, all participants had RSVPreF3-binding IgG concentrations above the LLOQ due to previous RSV exposure. The RSVPreF3-binding IgG concentration increased 11.84- (group 1), 11.29- (group 2), and 12.46-fold (group 3) 30 days post-vaccination compared to baseline (Table 2).

**Table 2:** RSVPreF3-binding IgG response at baseline and 30 days post-vaccination, per-protocol set.

**Variable**	**Group 1**	**Group 2**	**Group 3**
**Baseline (day 1)**			
N	248	251	250
GMC (95 % CI), EU/mL	7,332.9 (6,659.4–8,074.5)	7,290.1 (6,643.4–7,999.7)	7,520.9 (6,862.7–8,242.3)

**Post-vaccination (day 31)**			
N	234	237	237
GMC (95 % CI), EU/mL	86,039.9 (78,541.5–94,254.3)	80,518.0 (73,150.0–88,628.2)	94,260.9 (86,042.2–103,264.7)
MGI (95 % CI)	11.84 (10.53–13.31)	11.29 (10.12–12.60)	12.46 (11.13–13.94)

RSVPreF3, respiratory syncytial virus prefusion F protein; IgG, immunoglobulin G; N, number of participants with available data; GMC, geometric mean concentration; CI, confidence interval; EU, enzyme-linked immunosorbent assay unit; MGI, mean geometric increase of IgG concentration at day 31 compared to day 1.

### Reactogenicity and safety

**Fig. 4:**
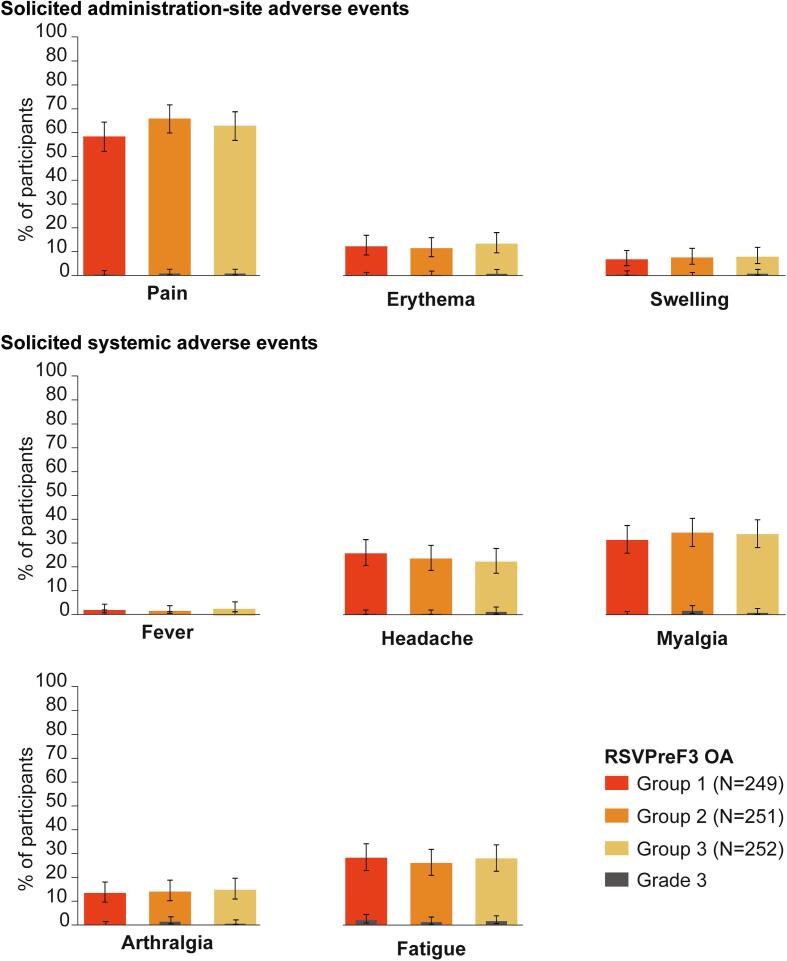
Incidence of solicited adverse events with onset within four days after vaccination, exposed set. N, number of participants with solicited safety data available; %, percentage of participants in a given category. Error bars depict 95 % confidence intervals. Grade 3: >100 mm (erythema and swelling); >39.0 °C (fever); symptom that prevents normal everyday activities (pain, headache, myalgia, arthralgia, fatigue).

In total, 73.1 % of participants reported at least one solicited AE with onset within four days of vaccination: 69.9 % (group 1), 75.3 % (group 2), and 74.2 % (group 3) of participants reported at least one solicited AE. Grade 3 solicited AEs were reported by 3.2 % (group 1), 3.6 % (group 2), and 4.4 % (group 3) of participants (Fig. 4**,** Supplementary table 1).

Most solicited administration-site AEs were mild or moderate and resolved within the four-day follow-up period (median duration of ≤ 3 days across the groups for AEs of any grade, and ≤ 2.5 days for grade 3 AEs). The most common solicited administration-site AE in each group was pain, with incidences of 58.2 % (group 1), 65.7 % (group 2), and 62.7 % (group 3). Grade 3 pain was reported in 0.4 % (group 1), 0.8 % (group 2), and 0.8 % (group 3) of participants.

Most solicited systemic AEs were mild or moderate and transient (median duration of ≤ 2 days across the groups for AEs of any grade and for grade 3 AEs). Myalgia was the most common solicited systemic AE, reported in 31.3 % (group 1), 34.3 % (group 2), and 33.7 % (group 3) of participants, followed by fatigue, reported in 28.1 % (group 1), 25.9 % (group 2), and 27.8 % (group 3) of participants. Grade 3 myalgia was reported in 1.6 % (group 2) and 0.8 % (group 3) of participants (none in group 1), and grade 3 fatigue in 2.0 % (group 1), 1.2 % (group 2), and 1.6 % (group 3) of participants.

In total, 14.3 % of participants reported at least one unsolicited AE with onset within 30 days of vaccination: 14.7 % (group 1), 14.6 % (group 2), and 13.4 % (group 3) of participants reported at least one unsolicited AE. Grade 3 unsolicited AEs were reported by 2.0 % (group 1), 2.4 % (group 2), and 1.6 % (group 3) of participants. At least one unsolicited AE that was considered by the investigator as related to the study vaccine was reported by 4.8 % (group 1), 5.9 % (group 2), and 4.7 % (group 3) of participants (Table 3).

**Table 3:** Incidence of unsolicited and serious adverse events, and potential immune-mediated diseases after vaccination, exposed set.

	Group 1		Group 2		Group 3	
	**N = 251**		**N = 253**		**N = 253**	
	**n**	**% (95 % CI)**	**n**	**% (95 % CI)**	**n**	**% (95 % CI)**
Unsolicited AE (30 days post-vaccination)						
Any	37	14.7 (10.6–19.7)	37	14.6 (10.5–19.6)	34	13.4 (9.5–18.3)
Grade 3	5	2.0 (0.6–4.6)	6	2.4 (0.9–5.1)	4	1.6 (0.4–4.0)
Vaccine-related*	12	4.8 (2.5–8.2)	15	5.9 (3.4–9.6)	12	4.7 (2.5–8.1)
Vaccine-related grade 3*	1	0.4 (0.0–2.2)	3	1.2 (0.2–3.4)	0	0.0 (0.0–1.4)

SAE (6 months post-vaccination)						
Any	8	3.2 (1.4–6.2)	6	2.4 (0.9–5.1)	7	2.8 (1.1–5.6)
Vaccine-related*	1	0.4 (0.0–2.2)	0	0.0 (0.0–1.4)	0	0.0 (0.0–1.4)
Fatal	0	0.0 (0.0–1.5)	2	0.8 (0.1–2.8)	2	0.8 (0.1–2.8)

pIMD (6 months post-vaccination)						
Any	2	0.8 (0.1–2.8)	1	0.4 (0.0–2.2)	3	1.2 (0.2–3.4)
Vaccine-related*	0	0.0 (0.0–1.5)	0	0.0 (0.0–1.4)	1	0.4 (0.0–2.2)

* By investigator assessment.

N, number of participants in the exposed set; n/%, number/percentage of participants in a given category; CI, confidence interval; AE, adverse event; SAE, serious adverse event; pIMD, potential immune-mediated disease.

Grade 3: A symptom that prevents normal everyday activities.

Until study end, at least one SAE was reported by 21 (2.8 %) participants: 3.2 % (group 1), 2.4 % (group 2), and 2.8 % (group 3) (Supplementary table 2). One SAE was considered by the investigator as related to the vaccine (atrial fibrillation, which started 162 days after vaccination; the participant was admitted to the hospital due to atrial fibrillation but was also diagnosed with probable COVID-19 pneumonia). SAEs with fatal outcomes were reported in a total of 4 (0.5 %) participants: 2 (0.8 %) in group 2, 2 (0.8 %) in group 3, and none in group 1 (Table 3). Fatal events included myocardial infarction, sudden cardiac death, chronic obstructive pulmonary disease exacerbation/pleural effusion/pulmonary edema, and cardiac arrest. None of these events were considered by the investigator as related to the vaccine.

In total, 6 (0.8 %) participants reported at least one pIMD: 2 (0.8 %) in group 1, 1 (0.4 %) in group 2, and 3 (1.2 %) in group 3. One of these was considered by the investigator as related to the vaccine (non-serious AE of worsening of pre-existing psoriasis, which started 14 days after vaccination and resolved after 167 days) (Table 3). A detailed description of all pIMDs is provided in Supplementary table 3.

## Discussion

In this phase 3, randomized, multicenter study, we evaluated the lot-to-lot immunogenicity consistency of three lots of RSVPreF3 OA in terms of RSVPreF3-binding IgG concentrations, as well as the vaccine’s reactogenicity and safety, in older adults. The primary confirmatory lot-to-lot consistency objective of the study was met, as the 95 % CIs of the RSVPreF3-binding IgG GMC ratios between lot pairs 30 days post-vaccination were within the pre-defined range (0.67–1.50). A descriptive evaluation of RSVPreF3-binding IgG responses after vaccination further confirmed the robust and comparable humoral immune response induced by the three lots 30 days post-vaccination.

The three lots were well tolerated, and the vaccine showed an acceptable safety profile. The reactogenicity profile was comparable across the three lot groups, and the reactions were mostly mild or moderate. The most frequently reported solicited AEs were administration-site pain, myalgia, and fatigue. Reactions were mostly transient, with a median duration of three days or less for administration-site AEs and two days or less for systemic AEs. The reported rates of SAEs and pIMDs were balanced between the three groups. One SAE (atrial fibrillation) was considered by the investigator as vaccine-related. This event started 162 days after vaccination, and the participant was also diagnosed with probable COVID-19 pneumonia upon hospitalization. One non-serious pIMD case of worsening of pre-existing psoriasis, which started 14 days after vaccination and eventually resolved, was considered by the investigator as vaccine-related. Overall, the safety results of the present study were comparable with those previously reported from other phase 3 RSVPreF3 OA studies [15], [16] and confirmed that the vaccine had an acceptable safety profile.

A strength of this study is that it was well powered to show immunological consistency of the vaccine lots in the vaccine target population, and the unique combination of both the antigen and adjuvant lots to the different vaccine lot groups adds to the validity of our findings.

In conclusion, this study demonstrated lot-to-lot consistency of three lots of the RSVPreF3 OA vaccine, which induced robust immune responses after one dose. The RSVPreF3 OA vaccine had an acceptable safety profile that was comparable between lots.

## Trademark statement

AS01 and *Arexvy* are trademarks owned by or licensed to GSK.

## Funding

This work was supported by GlaxoSmithKline Biologicals SA. GlaxoSmithKline Biologicals SA was involved in all stages of the study conduct and analysis and took responsibility for all costs associated with the development and the publishing of the present manuscript.

## CRediT authorship contribution statement

**Murdo Ferguson:** Data curation, Investigation, Writing – review & editing. **Alexander Murray:** Data curation, Formal analysis, Writing – review & editing, Investigation. **Lew Pliamm:** Data curation, Investigation, Writing – review & editing. **Lars Rombo:** Data curation, Investigation, Writing – review & editing. **Johan Sanmartin Berglund:** Data curation, Investigation, Writing – review & editing. **Marie-Pierre David:** Conceptualization, Formal analysis, Writing – review & editing. **Nathalie De Schrevel:** Conceptualization, Formal analysis, Writing – review & editing. **Franck Maschino:** Formal analysis, Writing – review & editing. **Shady Kotb:** Conceptualization, Formal analysis, Writing – review & editing. **Aurélie Olivier:** Conceptualization, Formal analysis, Writing – review & editing. **Veronica Hulstrøm:** Conceptualization, Formal analysis, Writing – review & editing.

## Declaration of competing interest

The authors declare the following financial interests/personal relationships which may be considered as potential competing interests: All authors reports financial support was provided by GlaxoSmithKline Biologicals SA. Alexander Murray (AM) reports a relationship with PharmQuest that includes: employment. AM reports that payments were made by GSK to his institution as clinical research trial site. If there are other authors, they declare that they have no known competing financial interests or personal relationships that could have appeared to influence the work reported in this paper.

All authors reports financial support was provided by GlaxoSmithKline Biologicals SA. Aurelie Olivier (AO) reports a relationship with GSK that includes: employment and equity or stocks. Aurelie Olivier (AO) has patent pending to GSK. AO is an employee of GSK at the time the study was designed, initiated, and/or conducted. AO holds shares of stock in the company as part of their employee remuneration. AO is co-applicants on a pending patent filed by GSK. If there are other authors, they declare that they have no known competing financial interests or personal relationships that could have appeared to influence the work reported in this paper.

All authors reports financial support was provided by GlaxoSmithKline Biologicals SA. Franck Maschino (FM) reports a relationship with GSK that includes: employment and equity or stocks. FM is an employee of GSK at the time the study was designed, initiated, and/or conducted. FM holds shares of stock in the company as part of their employee remuneration. If there are other authors, they declare that they have no known competing financial interests or personal relationships that could have appeared to influence the work reported in this paper.

All authors reports financial support was provided by GlaxoSmithKline Biologicals SA. Johan Sanmartin Berglund (JSB) reports a relationship with Blekinge Institute of Technology that includes: employment. Johan Sanmartin Berglund (JSB) has nothing else to disclose. If there are other authors, they declare that they have no known competing financial interests or personal relationships that could have appeared to influence the work reported in this paper.

All authors reports financial support was provided by GlaxoSmithKline Biologicals SA. Lew Pliamm (LP) reports a relationship with Canadian Phase Onward Inc. that includes: employment. LP has nothing else to disclose. If there are other authors, they declare that they have no known competing financial interests or personal relationships that could have appeared to influence the work reported in this paper.

All authors reports financial support was provided by GlaxoSmithKline Biologicals SA. Lars Rombo (LR) reports a relationship with Clinical Research Centre Sörmland that includes: employment. LR reports that payment was made by GSK to his institution for conducting the study. If there are other authors, they declare that they have no known competing financial interests or personal relationships that could have appeared to influence the work reported in this paper.

All authors reports financial support was provided by GlaxoSmithKline Biologicals SA. Murdo Ferguson (MF) reports a relationship with Colchester Research Group (CRG) that includes: employment. MF is employed by CRG which was contracted by GSK to execute the study. If there are other authors, they declare that they have no known competing financial interests or personal relationships that could have appeared to influence the work reported in this paper.

All authors reports financial support was provided by GlaxoSmithKline Biologicals SA. Marie-Pierre David (M-PD) reports a relationship with GSK that includes: employment and equity or stocks. Marie-Pierre David (M-PD) has patent pending to GSK. M-PD is an employee of GSK at the time the study was designed, initiated, and/or conducted. M-PD holds shares of stock in the company as part of their employee remuneration. M-PD is co-applicants on a pending patent filed by GSK. If there are other authors, they declare that they have no known competing financial interests or personal relationships that could have appeared to influence the work reported in this paper.

All authors reports financial support was provided by GlaxoSmithKline Biologicals SA. Nathalie De Schrevel (NDS) reports a relationship with GSK that includes: employment and equity or stocks. NDS is an employee of GSK at the time the study was designed, initiated, and/or conducted. NDS holds shares of stock in the company as part of their employee remuneration. If there are other authors, they declare that they have no known competing financial interests or personal relationships that could have appeared to influence the work reported in this paper.

All authors reports financial support was provided by GlaxoSmithKline Biologicals SA. Shady Kotb (SK) reports a relationship with GSK that includes: employment and equity or stocks. SK is an employee of GSK at the time the study was designed, initiated, and/or conducted. SK holds shares of stock in the company as part of their employee remuneration. If there are other authors, they declare that they have no known competing financial interests or personal relationships that could have appeared to influence the work reported in this paper.

All authors reports financial support was provided by GlaxoSmithKline Biologicals SA. Veronica Hulstrom reports a relationship with GSK that includes: employment and equity or stocks. VH is an employee of GSK at the time the study was designed, initiated, and/or conducted. VH holds shares of stock in the company as part of their employee remuneration. If there are other authors, they declare that they have no known competing financial interests or personal relationships that could have appeared to influence the work reported in this paper.

## Data Availability

Data will be made available on request. Anonymized data can be requested from http://www.clinicalstudydatarequest.com.
