# Supplementary material for: Lot-to-lot immunogenicity consistency of the respiratory syncytial virus prefusion F protein vaccine in older adults
Source: Vaccine X. 2024 Apr 27;18:100494. doi: 10.1016/j.jvacx.2024.100494 (PMC11090873; doi:10.1016/j.jvacx.2024.100494)
Supplement: Supplementary data 1 [file mmc1.pdf]

Lot-to-lot immunogenicity consistency of the respiratory syncytial virus prefusion F protein vaccine in older adults

Murdo Ferguson[a], Alexander Murray[b], Lew Pliamm[c], Lars Rombo[d], Johan Sanmartin Berglund[e], Marie-Pierre David[f], Nathalie De Schrevel[g], Franck Maschino[f], Shady Kotb[f]*, Aurélie Olivier[f], Veronica Hulstrøm[f]

[a]Colchester Research Group, 68 Robie, Truro, NS B2N 1L2, Canada

[b]PharmQuest, 806 Green Valley Rd Ste 305, Greensboro, NC 27408, United States

[c]Canadian Phase Onward Inc., Polyclinic Family and Specialty Medicine Facility, Polyclinic Family Health Group, 2 Champagne Dr, Toronto, ON M3J 0K2, Canada

[d]Clinical Research Centre Sörmland, Eskilstuna, SE-631 88, Sweden, and Department of Medical Biochemistry and Microbiology, Zoonosis Science Center, Uppsala University, Uppsala, SE-751 05, Sweden

[e]Department of Health, Blekinge Institute of Technology, Valhallavägen 1, Karlskrona, SE-371 79, Sweden, and Department of Clinical Sciences, Lund University, BMC I12, Lund, SE-221 84, Sweden

[f]GSK, Avenue Fleming 20, Wavre, 1300, Belgium

[g]GSK, Rue de l'Institut 89, Rixensart, 1330, Belgium

Supplementary materials

Supplementary methods

<u>Eligibility criteria</u>

Inclusion criteria

- Participants who, in the opinion of the investigator, could and would comply with the requirements of the protocol (e.g., completion of the diary cards, return for follow-up visit, ability to access and use a phone or other electronic communications).
  Note: In case of physical incapacity that would preclude the self-completion of the diary cards, either site staff could assist the participant (for activities performed during site visits) or the participant could assign a caregiver to assist him/her with this activity (for activities performed at home). However, at no time could the site staff or caregiver evaluate the participant's health status while answering diaries or make decisions on behalf of the participant.
- A male or female aged ≥60 years at the time of first study intervention administration.
- Participants living in the general community or in an assisted living facility that provided minimal assistance, such that the participant was primarily responsible for self-care and activities of daily living.
- Written or witnessed informed consent obtained from the participant prior to performance of any study-specific procedure.
- Participants who were medically stable in the opinion of the investigator at the time of vaccination. Participants with chronic stable medical conditions with or without specific treatment, such as diabetes, hypertension, or cardiac disease, could participate in this study if considered by the investigator as medically stable.

Exclusion criteria

Medical conditions

- Any confirmed or suspected immunosuppressive or immunodeficient condition resulting from disease (e.g., current malignancy, human immunodeficiency virus) or immunosuppressive/cytotoxic therapy (e.g., medication used during cancer chemotherapy, organ transplantation, or to treat autoimmune disorders), based on medical history and physical examination (no laboratory testing required).
- History of any reaction or hypersensitivity likely to be exacerbated by any component of the study intervention(s).
- Hypersensitivity to latex.
- Serious or unstable chronic illness.
- Any history of dementia or any medical condition that moderately or severely impairs cognition.

  Note: If deemed necessary for clinical evaluation, the investigator could use tools such as Mini Mental State Exam, Mini Cog or Montreal Cognitive Assessment to determine cognition levels of the participant.

- Recurrent or uncontrolled neurological disorders or seizures. Participants with medically controlled active or chronic neurological diseases could be enrolled in the study as per investigator assessment, provided that their condition would allow them to comply with the requirements of the protocol (e.g., completion of the diary cards, attend phone call/study site visits).
- Significant underlying illness that in the opinion of the investigator would be expected to prevent completion of the study (e.g., life-threatening disease likely to limit survival up to study end [i.e., six months post-vaccination]).

- Any medical condition that in the judgment of the investigator would make intramuscular injection unsafe.

Prior/concomitant therapy

- Use of any investigational or non-registered product (drug, vaccine, or medical device) other than the study intervention(s) during the period beginning 30 days before study intervention administration and ending 30 days after study intervention administration, or planned use during the study period.
- Planned or actual administration of a vaccine not foreseen by the study protocol in the period starting 30 days before and ending 30 days after the study intervention administration, with the exception of inactivated and subunit influenza vaccines, which could be administered up to 14 days before or from 14 days after the study vaccination. Note: In case an emergency mass vaccination for an unforeseen public health threat (e.g., a pandemic) was recommended and/or organized by the public health authorities, outside the routine immunization program, the time period described above could be reduced if necessary for that vaccine provided it was used according to the local governmental recommendations and that the Sponsor was notified accordingly.
- Previous vaccination with a respiratory syncytial virus (RSV) vaccine.
- Administration of long-acting immune-modifying drugs or planned administration at any time during the study period (e.g., infliximab).
- Administration of immunoglobulins and/or any blood products or plasma derivatives during the period starting 90 days before the administration of the study intervention or planned administration during the study period.
- Chronic administration (defined as more than 14 consecutive days in total) of immunosuppressants or other immune-modifying drugs during the period starting 90 days prior to the study intervention administration or planned administration during the study

period. For corticosteroids, this meant prednisone ≥20 mg/day or equivalent. Inhaled and topical steroids were allowed.

<u>Prior/concurrent clinical study experience</u>

- Concurrently participating in another clinical study, at any time during the study period, in which the participant had been or would be exposed to an investigational or a non-investigational vaccine/product (drug or invasive medical device).

<u>Other exclusions</u>

- History of chronic alcohol consumption and/or drug abuse as deemed by the investigator to render the potential participant unable/unlikely to provide accurate safety reports or comply with study procedures.
- Planned move during the study period that would prohibit participating in the study until study end.
- Bedridden participants.
- Participation of any study personnel or their immediate dependents, family, or household members.

<u>Study design</u>

Participants were enrolled with a balance between males and females, and in three age categories reflecting the age distribution in the general population, with approximately 40% of participants aged 60–69 years, 30% 70–79 years, and 10% ≥80 years. The remaining 20% were distributed freely across the age categories.

Randomization

Participants were randomized using an automated internet-based system, with a minimization method that accounted for age category (60–69 years, 70–79 years, and ≥80 years) and study center. Supplies were randomized within blocks for the different study centers, using the MATerial Excellence program, a program developed by GSK for use in SAS (Cary, NC, United States).

Intensity grading of solicited and unsolicited adverse events

All solicited and unsolicited AEs were graded as either 1 (mild), 2 (moderate), or 3 (severe).

Event	Grade	Parameter
**Administration-site solicited AE**		
Pain	1	Mild: any pain neither interfering with nor preventing normal activities
	2	Moderate: painful when limb is moved and interferes with normal activities
	3	Severe: significant pain at rest and prevents normal activities
Erythema/swelling	1	Mild: >20–≤50 mm
	2	Moderate: >50–≤100 mm
	3	Severe: >100 mm
**Systemic solicited AE**		
Fever	1	Mild: ≥38.0°C (100.4°F)–≤38.5°C (101.3°F)
	2	Moderate: >38.5°C (101.3°F)–≤39.0°C (102.2°F)
	3	Severe: >39.0°C (102.2°F)
Headache/fatigue/myalgia/arthralgia	1	Mild: present but easily tolerated
	2	Moderate: interferes with normal activities
	3	Severe: prevents normal activities

Unsolicited AE		
Any	1	Mild: present but easily tolerated
	2	Moderate: interferes with normal activities
	3	Severe: prevents normal activities

AE, adverse event.

Supplementary results

Supplementary table 1. Incidence of solicited adverse events with onset within four days after vaccination, exposed set

	Group 1 N=249		Group 2 N=251		Group 3 N=252	
**Solicited AE**	**n**	**% (95% CI)**	**n**	**% (95% CI)**	**n**	**% (95% CI)**
Any	174	69.9 (63.8–75.5)	189	75.3 (69.5–80.5)	187	74.2 (68.3–79.5)
Any Grade 3	8	3.2 (1.4–6.2)	9	3.6 (1.7–6.7)	11	4.4 (2.2–7.7)
**Administration-site AE**						
Any	149	59.8 (53.5–66.0)	168	66.9 (60.7–72.7)	162	64.3 (58.0–70.2)
Any Grade 3	2	0.8 (0.1–2.9)	3	1.2 (0.2–3.5)	5	2.0 (0.6–4.6)
**Pain**	145	58.2 (51.8–64.4)	165	65.7 (59.5–71.6)	158	62.7 (56.4–68.7)
Grade 3	1	0.4 (0.0–2.2)	2	0.8 (0.1–2.8)	2	0.8 (0.1–2.8)
**Erythema**	31	12.4 (8.6–17.2)	29	11.6 (7.9–16.2)	34	13.5 (9.5–18.3)
Grade 3	0	0.0 (0.0–1.5)	1	0.4 (0.0–2.2)	2	0.8 (0.1–2.8)
**Swelling**	17	6.8 (4.0–10.7)	19	7.6 (4.6–11.6)	20	7.9 (4.9–12.0)
Grade 3	1	0.4 (0.0–2.2)	0	0.0 (0.0–1.5)	2	0.8 (0.1–2.8)
**Systemic solicited AE**						

Any	113	45.4 (39.1–51.8)	127	50.6 (44.2–56.9)	119	47.2 (40.9–53.6)
Any Grade 3	6	2.4 (0.9–5.2)	7	2.8 (1.1–5.7)	7	2.8 (1.1–5.6)
**Fever**	5	2.0 (0.7–4.6)	4	1.6 (0.4–4.0)	7	2.8 (1.1–5.6)
Grade 3	1	0.4 (0.0–2.2)	0	0.0 (0.0–1.5)	0	0.0 (0.0–1.5)
**Headache**	64	25.7 (20.4–31.6)	59	23.5 (18.4–29.2)	56	22.2 (17.2–27.9)
Grade 3	1	0.4 (0.0–2.2)	1	0.4 (0.0–2.2)	3	1.2 (0.2–3.4)
**Myalgia**	78	31.3 (25.6–37.5)	86	34.3 (28.4–40.5)	85	33.7 (27.9–39.9)
Grade 3	0	0.0 (0.0–1.5)	4	1.6 (0.4–4.0)	2	0.8 (0.1–2.8)
**Arthralgia**	33	13.3 (9.3–18.1)	35	13.9 (9.9–18.9)	37	14.7 (10.6–19.7)
Grade 3	0	0.0 (0.0–1.5)	3	1.2 (0.2–3.5)	1	0.4 (0.0–2.2)
**Fatigue**	70	28.1 (22.6–34.1)	65	25.9 (20.6–31.8)	70	27.8 (22.3–33.7)
Grade 3	5	2.0 (0.7–4.6)	3	1.2 (0.2–3.5)	4	1.6 (0.4–4.0)

N, number of participants with solicited safety data available; AE, adverse event; n/%, number/percentage of participants in a given category; CI, confidence interval.

Grade 3: >100 mm (erythema and swelling); >39.0°C (fever); symptom that prevents normal activities (pain, headache, myalgia, arthralgia, fatigue).

Supplementary table 2. Participants with at least one serious adverse event reported from vaccination until study end, exposed set

Primary System Organ Class<br>Preferred Term	Group 1<br>N=251		Group 2<br>N=253		Group 3<br>N=253		Overall<br>N=757	
	n	% (95% CI)	n	% (95% CI)	n	% (95% CI)	n	% (95% CI)
Any	8	3.2 (1.4–6.2)	6	2.4 (0.9–5.1)	7	2.8 (1.1–5.6)	21	2.8 (1.7–4.2)
Cardiac disorders	1	0.4 (0.0–2.2)	2	0.8 (0.1–2.8)	3	1.2 (0.2–3.4)	6	0.8 (0.3–1.7)
Acute myocardial infarction	0	0.0 (0.0–1.5)	1	0.4 (0.0–2.2)	1	0.4 (0.0–2.2)	2	0.3 (0.0–1.0)
Myocardial infarction	0	0.0 (0.0–1.5)	1	0.4 (0.0–2.2)	0	0.0 (0.0–1.4)	1	0.1 (0.0–0.7)
Atrial fibrillation	1	0.4 (0.0–2.2)	0	0.0 (0.0–1.4)	1	0.4 (0.0–2.2)	2	0.3 (0.0–1.0)
Cardiac arrest	0	0.0 (0.0–1.5)	0	0.0 (0.0–1.4)	1	0.4 (0.0–2.2)	1	0.1 (0.0–0.7)
Infections and infestations	2	0.8 (0.1–2.8)	2	0.8 (0.1–2.8)	0	0.0 (0.0–1.4)	4	0.5 (0.1–1.3)
Large intestine infection	0	0.0 (0.0–1.5)	1	0.4 (0.0–2.2)	0	0.0 (0.0–1.4)	1	0.1 (0.0–0.7)
COVID-19	1	0.4 (0.0–2.2)	0	0.0 (0.0–1.4)	0	0.0 (0.0–1.4)	1	0.1 (0.0–0.7)
Pneumonia	0	0.0 (0.0–1.5)	1	0.4 (0.0–2.2)	0	0.0 (0.0–1.4)	1	0.1 (0.0–0.7)
Sepsis	1	0.4 (0.0–2.2)	0	0.0 (0.0–1.4)	0	0.0 (0.0–1.4)	1	0.1 (0.0–0.7)
Respiratory, thoracic, and mediastinal disorders	1	0.4 (0.0–2.2)	0	0.0 (0.0–1.4)	3	1.2 (0.2–3.4)	4	0.5 (0.1–1.3)

Pulmonary embolism	1	0.4 (0.0–2.2)	0	0.0 (0.0–1.4)	2	0.8 (0.1–2.8)	3	0.4 (0.1–1.2)
Chronic obstructive pulmonary disease	0	0.0 (0.0–1.5)	0	0.0 (0.0–1.4)	1	0.4 (0.0–2.2)	1	0.1 (0.0–0.7)
Pleural effusion	0	0.0 (0.0–1.5)	0	0.0 (0.0–1.4)	1	0.4 (0.0–2.2)	1	0.1 (0.0–0.7)
Pulmonary edema	0	0.0 (0.0–1.5)	0	0.0 (0.0–1.4)	1	0.4 (0.0–2.2)	1	0.1 (0.0–0.7)
Neoplasms benign, malignant, and unspecified	2	0.8 (0.1–2.8)	1	0.4 (0.0–2.2)	0	0.0 (0.0–1.4)	3	0.4 (0.1–1.2)
Squamous cell carcinoma of the hypopharynx	0	0.0 (0.0–1.5)	1	0.4 (0.0–2.2)	0	0.0 (0.0–1.4)	1	0.1 (0.0–0.7)
Metastases to liver	1	0.4 (0.0–2.2)	0	0.0 (0.0–1.4)	0	0.0 (0.0–1.4)	1	0.1 (0.0–0.7)
Metastases to lymph nodes	1	0.4 (0.0–2.2)	0	0.0 (0.0–1.4)	0	0.0 (0.0–1.4)	1	0.1 (0.0–0.7)
Pancreatic carcinoma	1	0.4 (0.0–2.2)	0	0.0 (0.0–1.4)	0	0.0 (0.0–1.4)	1	0.1 (0.0–0.7)
Prostate cancer	1	0.4 (0.0–2.2)	0	0.0 (0.0–1.4)	0	0.0 (0.0–1.4)	1	0.1 (0.0–0.7)
Metabolism and nutrition disorders	0	0.0 (0.0–1.5)	2	0.8 (0.1–2.8)	0	0.0 (0.0–1.4)	2	0.3 (0.0–1.0)
Gout	0	0.0 (0.0–1.5)	1	0.4 (0.0–2.2)	0	0.0 (0.0–1.4)	1	0.1 (0.0–0.7)
Malnutrition	0	0.0 (0.0–1.5)	1	0.4 (0.0–2.2)	0	0.0 (0.0–1.4)	1	0.1 (0.0–0.7)
Nervous system disorders	1	0.4 (0.0–2.2)	0	0.0 (0.0–1.4)	1	0.4 (0.0–2.2)	2	0.3 (0.0–1.0)

Ischemic stroke	1	0.4 (0.0–2.2)	0	0.0 (0.0–1.4)	0	0.0 (0.0–1.4)	1	0.1 (0.0–0.7)
Syncope	0	0.0 (0.0–1.5)	0	0.0 (0.0–1.4)	1	0.4 (0.0–2.2)	1	0.1 (0.0–0.7)
General disorders and administration-site conditions	0	0.0 (0.0–1.5)	1	0.4 (0.0–2.2)	0	0.0 (0.0–1.4)	1	0.1 (0.0–0.7)
Sudden cardiac death	0	0.0 (0.0–1.5)	1	0.4 (0.0–2.2)	0	0.0 (0.0–1.4)	1	0.1 (0.0–0.7)
Hepatobiliary disorders	1	0.4 (0.0–2.2)	0	0.0 (0.0–1.4)	0	0.0 (0.0–1.4)	1	0.1 (0.0–0.7)
Cholecystitis	1	0.4 (0.0–2.2)	0	0.0 (0.0–1.4)	0	0.0 (0.0–1.4)	1	0.1 (0.0–0.7)
Injury, poisoning, and procedural complications	1	0.4 (0.0–2.2)	0	0.0 (0.0–1.4)	0	0.0 (0.0–1.4)	1	0.1 (0.0–0.7)
Fall	1	0.4 (0.0–2.2)	0	0.0 (0.0–1.4)	0	0.0 (0.0–1.4)	1	0.1 (0.0–0.7)
Renal and urinary disorders	0	0.0 (0.0–1.5)	1	0.4 (0.0–2.2)	0	0.0 (0.0–1.4)	1	0.1 (0.0–0.7)
Urethral stenosis	0	0.0 (0.0–1.5)	1	0.4 (0.0–2.2)	0	0.0 (0.0–1.4)	1	0.1 (0.0–0.7)

N, number of participants in the exposed set; n/%, number/percentage of participants in a given category; CI, confidence interval.

Note: Study end: six months post-vaccination.

Supplementary table 3. Potential immune-mediated diseases reported from vaccination until study end, exposed set

Preferred term	Age onset (years)	Gender	Day onset	Recovered/ resolved	Duration (days)	Lot group	Country	Vaccine-related*	SAE	Additional notes
Gout	69	Male	182	Yes	6	1	Canada	No	No	
Gout	67	Male	65	Yes	3	1	Canada	No	No	
Gout	77	Male	158	Yes	40	2	Canada	No	Yes	
Rheumatoid arthritis	77	Male	112	No	-	3	Canada	No	No	
Psoriasis	79	Male	15	Yes	167	3	Canada	Yes	No	Worsening of pre-existing condition
Psoriasis	69	Female	3	No	-	3	Sweden	No	No	Worsening of pre-existing condition

*By investigator assessment.

SAE, serious adverse event.

Notes: Each of the potential immune-mediated diseases was reported in a different participant. Study end: six months post-vaccination.
